# Molecular profile of patients with myelofibrosis: a 10-year experience

**DOI:** 10.31744/einstein_journal/2023AO0100

**Published:** 2022-12-15

**Authors:** Lara Faria Souza Dias, Carolina Leme de Moura Pereira, Newton de Freitas Centurião, Jade Zezzi Martins do Nascimento, Andreza Alice Feitosa Ribeiro, Nelson Hamerschlak, Carolina Perrone Marques, Ana Carolina Vieira de Lima, Luana Nóbrega da Costa, Anderson Felipe da Silva, Viviane de Jesus Torres Lima, Mariana Nassif Kerbauy, Lucila Nassif Kerbauy, Leonardo Javier Arcuri, Paulo Vidal Campregher, Juliana Dall´Agnol da Rocha, Tarcila Santos Datoguia, Fabio Pires de Souza Santos

**Affiliations:** 1 Hospital Israelita Albert Einstein São Paulo SP Brazil Hospital Israelita Albert Einstein, São Paulo, SP, Brazil.

**Keywords:** Primary myelofibrosis, Cytogenetic analysis, Mutation, Prognosis, Bone marrow transplantation

## Abstract

**Objective:**

To analyze the karyotype test and myeloid panel with next-generation sequencing findings in patients with myelofibrosis, and to compare transplant characteristics in patients referred for bone marrow transplantation.

**Methods:**

Retrospective, single-center study with patients diagnosed with myelofibrosis treated at *Hospital Israelita Albert Einstein* between 2010 and 2020.

**Results:**

A total of 104 patients with myelofibrosis were examined. Patients who had not been submitted to tests in our service were excluded. The final sample comprised 69 patients. Of these 69, 56 were submitted to karyotyping and 22 to myeloid panel with next-generation sequencing. Karyotype was normal in 60% of the patients and altered in 40%. The prevalence of high-risk molecular mutations was higher in patients referred for bone marrow transplantation (100% *versus* 50%). The median follow-up of transplant patients was 2.4 years and the overall survival at 2 years was 80% (95%CI: 62-100%).

**Conclusion:**

The molecular analysis enables estimating the patient’s risk and thus instituting more aggressive treatment such as bone marrow transplant for patients at higher risk, being a relevant tool to guide therapy. Given the significance of molecular analysis for therapeutic decision-making in myelofibrosis, collection and disclosure of data on the prevalence of cytogenetic changes and findings of next-generation sequencing in affected patients is important.

## INTRODUCTION

Myelofibrosis (MF) is a BCR-ABL-1 negative myeloproliferative neoplasm. This type of neoplasm can be primary (“*de novo*” presentation) or secondary to polycythemia vera (PV) or essential thrombocythemia (ET).^(1,2)^ Primary MF is more prevalent and affects 4 to 6 people per 100,000 population, whereas post-ET and post PV MF affect 0.5 to 1.1 and 0.3 to 0.7 per 100,000 people respectively.^(3,4)^ Myelofibrosis is slightly more common in elderly males with median age at diagnosis of 64 years.^(5)^ The estimated median overall survival of patients with primary MF or MF secondary to PV is 4.5 years, compared to 7.06 years in patients with MF secondary to ET.^(1)^ The primary causes of death include leukemic transformation, cardiovascular events and complications of cytopenia, such as infection or bleeding.^(2)^

Myelofibrosis is associated with the presence of 3 cardinal and often mutually exclusive mutations: janus kinase 2 V617F (JAK2V617F), calreticulin (CALR) and myeloproliferative leukemia virus (MPL) oncogene.^(2)^ Only 10% of patients are triple negative (no JAK2, CALR or MPL mutations). These patients have lower survival rates and higher risk of progression to acute myeloid leukemia (AML).^(1)^Genetic markers are determinant of outcomes in patients with MF and have been incorporated into formal prognostic systems, such as MIPSS70+ and GIPSS.^(1)^Other risk factors which contribute to progression to AML include unfavorable karyotypes, circulating blast percentages higher than 3%, platelet counts less than 50,000, TP53, and high-risk somatic mutations such as ASXL1 (frequency of 22%), SRSF2 (9%), EZH2 (5%), IDH1/2 (3%) and U2AF1 Q157 (16%).^(1)^

Despite the availability of new therapeutic agents to tackle MF, the only treatment with curative potential is allogeneic bone marrow transplantation (BMT).^(2)^Unfortunately, BMT is associated with at least 50% of transplant-related deaths or severe morbidity such as graft-*versus*-host disease (GVHD) in MF patients.^(2)^ In a Mayo Clinic study with MF patients submitted to allogeneic BMT, the 5-year overall survival was 62%.^(6)^ Hence, risks associated with BMT must be weighed according to life expectancy, should the patient not receive BMT. For this type of assessment, molecular genetic risk factors of patients must be determined.^(2)^

## OBJECTIVE

To analyze the karyotype test and myeloid panel with next-generation sequencing findings in patients with myelofibrosis, and to compare transplant characteristics in patients referred for bone marrow transplantation.

## METHODS

### Study design

Retrospective, single-center study based on medical records of patients diagnosed with MF treated at *Hospital Israelita Albert Einstein* (HIAE) between 2010 and 2020. Patient data (age, sex, clinical status, date of diagnosis, karyotype, mutations, treatments, last follow-up, and death) were collected, as well as transplant data of patients undergoing BMT (conditioning regimen, cell source, type of transplant, neutrophil engraftment, and occurrence of GVHD).

### Inclusion criteria

Patients diagnosed with myelofibrosis treated at HIAE between 2010 and 2020. Primary and secondary MF diagnosis were defined according to 2016 World Health Organization (WHO) diagnostic criteria.^(1)^

Patients with available clinical, laboratory and therapeutic data in medical records.

### Exclusion criteria

Inability to retrospectively collect patient and clinical outcome data.

### Definitions and outcomes

Overall survival was defined as survival from the date of diagnosis to the date of death from any cause. Patients who were alive at the time of analysis were censored.

### Statistical analysis

Overall survival was estimated using the Kaplan-Meier method. Categorical and continuous variables were reported using descriptive statistics. Statistical tests were performed using R software, version 4.0.0.

### Approval by the ethics committee

This project was approved by the Ethics Committee of HIAE. Certificate of submission to ethical assessment CAAE: 47080621.1.0000.0071; # 5.208.295.

## RESULTS

**Figure 1:**
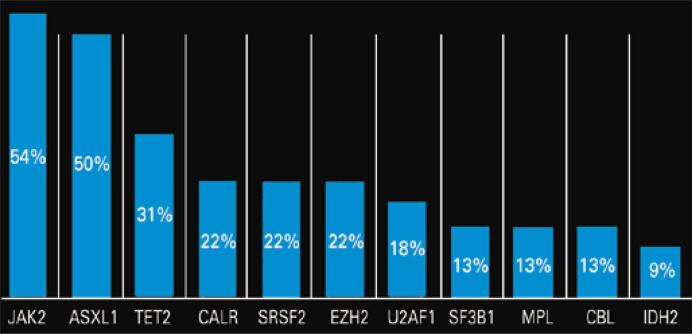
Prevalence of mutations in next-generation sequencing panels (22 patients)

JAK2: janus kinase 2 V617F; ASXL1: ASXL transcriptional regulator 1; TET2: TET methylcytosine dioxygenase 2; CALR: calreticulin; SRSF2: serine/arginine-rich splicing factor 2; EZH2: enhancer of zeste homolog 2; U2AF1: U2 small nuclear RNA auxiliary factor 1; SF3B1: splicing factor 3B subunit 1; MPL: myeloproliferative leukemia virus oncogene; CBL: casitas B-cell lineage lymphoma; IDH2: isocitrate dehydrogenase 2.

**Figure 2:**
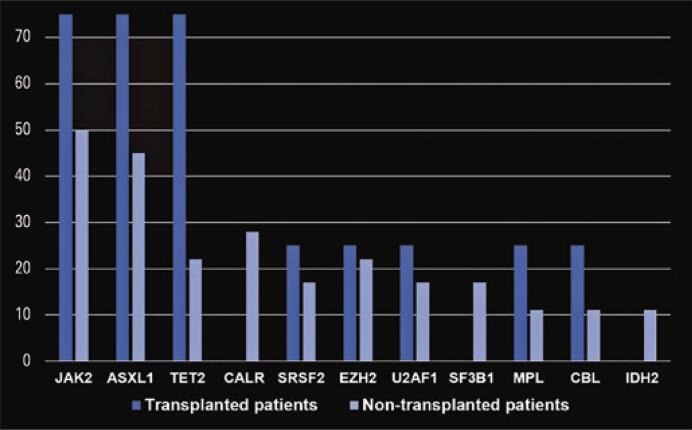
Prevalence of mutations found in the next-generation sequencing panels of transplanted and non-transplanted patients

JAK2: janus kinase 2 V617F; ASXL1: ASXL transcriptional regulator 1; TET2: TET methylcytosine dioxygenase 2; CALR: calreticulin; SRSF2: serine/arginine-rich splicing factor 2; EZH2: enhancer of zeste homolog 2; U2AF1: small nuclear RNA auxiliary fator 1; SF3B1: splicing factor 3B subunit 1; MPL: myeloproliferative leukemia virus oncogene; CBL: casitas B-cell lineage lymphoma; IDH2: isocitrate dehydrogenase 2.

A total of 104 patients with MF were evaluated. Patients who had not been tested at our service were excluded. The final sample comprised 69 patients. Of these 69, 56 were submitted to karyotyping and 22 to myeloid panel with NGS. The karyotype was normal in 60% of patients and altered in 40%. The most prevalent changes were: trisomy 8 (22%), deletion of 20 (22%), deletion of 5 (18%), deletion of 9 (13%), trisomy 9 (13%) and monosomy 7 and 17 (9% each). In the 22 patients examined using NGS, the following variants were detected: JAK2 (54%) ASXL1 (50%), TET2 (31%), CALR (22%), SRSF2 (22%), EZH2 (22%), U2AF1 (18%), SF3B1 (13%), MPL (13%), CBL (13%), IDH2 (9%) (Figure 1). The prevalence of high-risk molecular mutations (ASXL1, SRSF2, EZH2, IDH1/2 and U2AF1 Q157P) was higher in patients referred for BMT (100% v*ersus* 50%) (Figure 2).

Sixteen patients underwent BMT. Median follow-up was 2.4 years, with a 2-year overall survival of 80% (95%CI: 62-100%). Of these 16 patients, 11 were women (68%) with median age at diagnosis of 54 years and median age at the time of BMT of 57 years. Median time from diagnosis to BMT was 5.6 years (1 to 19 years). Half of patients were asymptomatic at diagnosis and had been referred for investigation of changes in blood cell count. At diagnosis, 8 patients had splenomegaly (50%), 4 had night sweats (25%) and 2 had lost weight (12.5%). Of the 14 patients amenable to cytogenetic assessment, 11 were normal (78%) and 3 had changes (21%, del5, n=2; del20, n=1). Ten patients carried the JAK2V617F mutation (83%), 1 carried the CALR mutation and 1 carried the MPL mutation (8%). Other mutations detected were ASXL1 (33%), SRSF2 (16%), TET2 (16%), ETV6, EZH2 and U2AF1. Primary MF accounted for 60% of cases, whereas MF secondary to ET was diagnosed in 40% of patients.

Out of 16 transplants performed, 5 were matched-sibling, 5 were matched unrelated 10/10 human leukocyte antigen (HLA) donors, 2 were mismatched unrelated, 1 was cord blood, and were 3 haploidentical (Table 1). Seven patients (43%) had been previously treated with the JAK2 inhibitor, ruxolitinib. Peripheral blood cell source was used in 9 patients (56.25%), followed by bone marrow (37.5%) and umbilical cord blood (1 case, 6.25%). Reduced-intensity and myeloablative transplantation were performed in 9 (56%) and 7 patients (44%), respectively. Busulfan-fludarabine ± antithymocyte globulin (BuFlu±ATG) was the most common conditioning regimen. Serum levels of busulfan were calculated from the 4,000 AUC of ATG in cases with unrelated donors (43.75%). All patients achieved neutrophil engraftment within a median time of 15 days (9-22). Six patients died. Causes of death were as follows: cerebral ischemia (n=1), infection (n=1) and transformation to acute myeloid leukemia (n=1). Of the 16 patients, 2 developed grade IV (12.5%), 3 developed grade III (18.75%) and 4 developed grade II (25%) acute GVHD.

**Table 1:** Type of transplantation performed and number of deaths per subtype

Type of BMT	Number of patients	Number of deaths
Related 10x10 HLA matching	5	1
Unrelated 10x10 HLA matching	5	3
Haploidentical	3	1
Related with mismatch	2	2
Cord blood	1	0

BMT: bone marrow transplantation; HLA: human leukocyte antigen.

Of 16 transplants performed, 5 were matched-sibling, 5 unrelated 10x10, 2 unrelated with mismatch, 1 cord blood and 3 haploidentical.

## DISCUSSION

One of the major causes of death in patients with MF is leukemic transformation, which occurs in 20% of cases.^(3)^The main risk factors for transformation into AML are unfavorable karyotypes, such as monosomies or inv(3)/i(17q), and high-risk somatic mutations, such as ASXL1 (population frequency 22%), SRSF2 (9%), EZH2 (5%), IDH1/2 (3%) and U2AF1 Q157 (16%).^(1)^Findings of three clinical trials suggest patients carrying the ASXL1 mutation respond less to JAK2 inhibitors, while patients carrying the CALR type 1 mutation show longer-lasting responses.^(4)^These mutations affect patient survival and response to treatment.

The prevalence of high-risk somatic mutations in patients submitted to NGS in this sample was higher than the prevalence reported in the general population (Table 2).^(1)^ This may have reflected that, in this study, NGS panels were used to help decide whether patients at higher risk should be referred for BMT.

**Table 2:** Prevalence of somatic mutations found in the NGS panel of myelofibrosis patients examined at HIAE and in other studies

Somatic mutations	ASXL1	U2AF1	SRSF2	EZH2	IDH1/2
HIAE population (%)	50	18	22	22	9
General population (%)	22	16	9	5	3

HIAE: *Hospital Israelita Albert Einstein*; ASXL1: ASXL transcriptional regulator 1; U2AF1: small nuclear RNA auxiliary factor 1; SRSF2: serine/arginine-rich splicing factor 2; EZH2: enhancer of zeste homolog 2; IDH2: isocitrate dehydrogenase 2.

Most drugs available for MF treatment are palliative and aimed to alleviate symptoms, reduce complications and improve quality of life, with no impact on the natural history of disease or survival.^(2)^The only potentially curative treatment is BMT, which has a high morbidity and mortality.^(3)^

In spite of the small number of patients undergoing BMT in this study, results were consistent with findings reported in prior studies using the BuFlu regimen with appropriate busulfan doses. Optimization of conditioning regimens and use of JAK2 inhibitors are thought to improve engraftment rates in BMT for myelofibrosis. However, new strategies are needed to reduce GVHD incidence and post-transplantation relapse rates, to improve clinical outcomes of transplanted patients.

Gowin et al. compared the survival of 1,928 patients with myelofibrosis submitted to allogeneic BMT (551) or clinical treatment (1,377).^(7)^Patients undergoing allogeneic transplantation had shorter 1-year survival relative to patients receiving clinical treatment. However, over the course of 6-year follow-up, MF patients treated with BMT who had intermediate-1 or higher-risk DIPSS had better long-term survival, despite higher early mortality rates.^(7)^ Low-risk patients did not benefit from BMT and had poorer survival rates compared to patients undergoing clinical treatment.

## CONCLUSION

Optimization of conditioning regimens and use of JAK2 inhibitors are thought to improve engraftment rates in bone marrow transplantation for myelofibrosis. Ruxolitinib has important immunosuppressive effects, and may help control graft-*versus*-host disease after allogeneic bone marrow transplantation. However, new strategies are needed to reduce the incidence of graft-*versus*-host disease and improve clinical outcome of these transplanted patients. Karyotype analysis and graft-*versus*-host disease can be used to estimate a patient’s risk. These approaches may inform the need of more aggressive treatment in higher risk patients, such as bone marrow transplantation. Molecular analysis is thought to be a relevant tool to guide therapy.
